# Delivery after Death—Life of a Six Months’ Fœtus

**Published:** 1875-04

**Authors:** 


Delivery after Death—Life of a Six Months' Fœtus.—Rigaudeaux (Meigs' Velpeau, p. 509) relates that he was sent for, two leagues from Douai, to see a woman whose labor had excited great uneasiness; when he arrived she was believed to have been dead for two hours. Instead of opening the abdomen without an examination, he explored the genital organs, found the pelvis well formed, and proceeded to turn and deliver the child by the feet. It was born in a state of apparent death, but with great exertion was brought to life in about two hours. The limbs of the mother preserving their suppleness, he forbade them to bury her until the abdomen should have turned green. After a few hours this woman recovered so completely from her insensibility that she came herself, four years afterwards, to inform Rigaudeaux that she was not dead.

Dr. Atlee mentioned an instance which occurred in his practice, in December, 1845. By the closest calculation the period of gestation had not exceeded six months. A very small child was born. It being very flaccid and apparently lifeless, it was rolled carefully in a cloth and laid to one side. After having recovered the placenta and placed the mother comfortably in bed, he was asked the sex of the child. Unwrapping it, he noticed a slight gasp, which was followed by others. It was now laid in a bed of cotton wadding, and its grandmother took charge of it, feeding it by dropping milk into its mouth from the point of her finger. It was kept in this bed of cotton and fed in this way for two weeks, when it was first washed and dressed. At that time it weighed two and a fourth pounds. It continued to live, and is now a beautiful and vigorous young lady.—*Philadelphia Medical Times.*

Labor and Longevity.—Work by method and on system, even when severe, is not only quite compatible with prolonged life, but is actually conducive to it, while the torpor of idleness or the excitement of fitful effort are the sure precursors of senile degeneration. This is a useful doctrine to preach, and still more useful to practise.—*Lancet.*

The difference between having a tooth properly drawn by a professional surgeon, and having it knocked out miscellaneously by a fall on the pavement is only a slight distinction—one is dental and the other acci-dental.

